# The Effect of Antifibrotic Drugs in Rat Precision-Cut Fibrotic Liver Slices

**DOI:** 10.1371/journal.pone.0095462

**Published:** 2014-04-22

**Authors:** Inge M. Westra, Dorenda Oosterhuis, Geny M. M. Groothuis, Peter Olinga

**Affiliations:** 1 Pharmacokinetics, Toxicology and Targeting, Department of Pharmacy, University of Groningen, Groningen, The Netherlands; 2 Pharmaceutical Technology and Biopharmacy, Department of Pharmacy, University of Groningen, Groningen, The Netherlands; Haassah Medical Center, Israel

## Abstract

Two important signaling pathways in liver fibrosis are the PDGF- and TGFβ pathway and compounds inhibiting these pathways are currently developed as antifibrotic drugs. Testing antifibrotic drugs requires large numbers of animal experiments with high discomfort. Therefore, a method to study these drugs *ex vivo* was developed using precision-cut liver slices from fibrotic rat livers (fPCLS), representing an *ex vivo* model with a multicellular fibrotic environment. We characterized the fibrotic process in fPCLS from rat livers after 3 weeks of bile duct ligation (BDL) during incubation and tested compounds predominantly inhibiting the TGFβ pathway (perindopril, valproic acid, rosmarinic acid, tetrandrine and pirfenidone) and PDGF pathway (imatinib, sorafenib and sunitinib). Gene expression of heat shock protein 47 (*Hsp47*), α smooth muscle actin (*αSma*) and pro-collagen 1A1 (*Pcol1A1*) and protein expression of collagens were determined. During 48 hours of incubation, the fibrosis process continued in control fPCLS as judged by the increased gene expression of the three fibrosis markers, and the protein expression of collagen 1, mature fibrillar collagen and total collagen. Most PDGF-inhibitors and TGFβ-inhibitors significantly inhibited the increase in gene expression of *Hsp47, αSma* and *Pcol1A1.* Protein expression of collagen 1 was significantly reduced by all PDGF-inhibitors and TGFβ-inhibitors, while total collagen was decreased by rosmarinic acid and tetrandrine only. However, fibrillar collagen expression was not changed by any of the drugs. In conclusion, rat fPCLS can be used as a functional *ex vivo* model of established liver fibrosis to test antifibrotic compounds inhibiting the PDGF- and TGFβ signalling pathway.

## Introduction

During liver fibrosis, connective tissue accumulates progressively and affects the normal function of the liver. The hepatic stellate cells (HSC) play a pivotal role in the development of liver fibrosis. Upon chronic injury, HSC are activated and transdifferentiate into myofibroblasts that have fibrogenic properties and are the main producers of collagen [1], [2].

During fibrosis, different signaling pathways are activated. The two most important pathways in liver fibrosis are the platelet-derived growth factor (PDGF)- and the transforming growth factor beta (TGFβ) signaling pathway. Activation of these pathways results in proliferation of myofibroblasts and excess deposition of collagen [3]–[5]. Therefore many compounds inhibiting one of these pathways have been developed as potential antifibrotic drugs, some of which entered clinical studies [6]. However no effective medicines against end-stage liver fibrosis are available yet.

PDGF is the most important proliferative factor for HCS and myofibroblasts in liver fibrogenesis. During transition of quiescent HSC into activated HSC with a myofibroblast phenotype, they release PDGF. This PDGF binds to the PDGF receptor on activated HCS and activates the PDGF pathway, but not in quiescent HSC, as they do not express the PDGF receptor [7]. In addition, Kupffer cells and hepatocytes can increase the release of PDGF and the expression of the PDGF receptor in HSC [8]. Furthermore, after HSC activation and differentiation, TGFβ, produced by hepatocytes and Kupffer cells induces a growth stimulatory effect in transdifferentiated myofibroblasts, resulting in extracellular matrix deposition [9].

In order to study the mechanism of fibrosis and the effect of antifibrotic compounds, several *in vitro* models have been developed. The use of precision-cut tissue slices as *ex vivo* model to study fibrosis in different organs has recently been reviewed [10]. The major advantages of the use of precision-cut tissue slices are the presence of the intact organ architecture, cell-cell and cell-matrix interactions and the potential to use human tissue and to contribute to a large reduction in the use of laboratory animals for testing antifibrotic drugs [11], [12]. Recently, the early onset of liver fibrosis was investigated using rat precision-cut liver slices (PCLS) [13], [14]. Long-term culture for 48 hours of PCLS, prepared from livers from healthy rats, induced activation of HSC and induction of fibrosis markers, which could be inhibited by several antifibrotic compounds acting on the PDGF- signaling pathway but not by compounds acting via the TGFβ pathway [14].

The aim of the present study was to investigate whether PCLS from livers of rats with established fibrosis (fPCLS) can be used to investigate the antifibrotic effects of drugs. Previously we reported that fPCLS from bile-duct ligated (BDL) rats with established fibrosis showed progression of the fibrosis process during incubation which could be inhibited by pentoxifylline, imatinib and dexamethasone [15]. Moreover it was shown that during culture up to 48 hours, both parenchymal and non-parenchymal cells in fPCLS from BDL rats remained functionally active. In the present study, we investigated the efficacy of a series of antifibrotic compounds inhibiting the PDGF- or the TGFβ pathway in fPCLS from BDL rats. The PDGF-inhibitors imatinib, sorafenib and sunitinib are tyrosine kinase inhibitors that have antifibrotic effects *in vitro* and *in vivo* in rats [16]–[18]. The TGFβ-inhibitors perindopril, an angiotensin converting enzyme (ACE) inhibitor, valproic acid, a histone deacetylase inhibitor, rosmarinic acid and pirfenidone, antifibrotic compounds that inhibit the TGFβ expression, and tetrandrine, which up-regulates smad7, also demonstrated antifibrotic effects *in vitro* and *in vivo* in liver fibrosis [19]–[24]. In addition, we also tested colchicine, which antifibrotic effects were shown in HSC and cirrhotic rats [25]. Based on the results, we conclude that fPCLS are an adequate model to test the efficacy of antifibrotic compounds.

## Materials and Methods

### Ethics statement

Adult male Wistar rats (Ctrl:WI) were purchased from Charles River (Sulzfeld, Germany).

The rats were housed on a 12 hours light/dark cycle in a temperature-and-humidity-controlled room with food (Harlan chow no 2018, Horst, The Netherlands) and water ad libitum. The animals were allowed to acclimatize for at least seven days before the start of the experiments. The experiments were approved by the Animal Ethical Committee of the University of Groningen. The rats were anaesthetized with isoflurane (Nicholas Piramal, London, UK) and subjected to BDL [26], and all efforts were made to minimize suffering.

### Slice experiments

Three weeks after BDL, livers were harvested and used for preparing liver slices in ice-cold Krebs-Henseleit buffer supplemented with 25 mM D-glucose (Merck, Darmstadt, Germany), 25 mM NaHCO_3_ (Merck), 10 mM Hepes (MP Biomedicals, Aurora, OH, USA) and saturated with carbogen (95% O_2_/5% CO_2_) using a Krumdieck tissue slicer [11]. fPCLS with a diameter of 5 mm and a thickness of 250 µm were incubated up to 48 hours in 1.3 ml of Williams Medium E (with L-glutamine, Invitrogen, Paisly, Scotland) supplemented with 25 mM glucose and 50 µg/ml gentamycin (Invitrogen), with the antifibrotic compounds, at 37°C and 95% O_2_/5% CO_2_ in 12-wells plates while gently shaken [11]. As a control, slices were incubated for 1, 24 and 48 hours with the solvent. After 24 hours the slices were transferred to new 12 wells plates with fresh medium. The slices were incubated with the antifibrotic compounds imatinib (1–10 µM) (Novartis, Basel, Switzerland), valproic acid (0.1–1 mM) (Sigma Aldrich, Zwijndrecht, Netherlands), perindopril (10–100 µM) (Sigma Aldrich), pirfenidone (0.5–2.5 mM) (Sigma Aldrich), rosmarinic acid (120–270 µM) (Sigma Aldrich), colchicine (30–200 nM) (Sigma Aldrich), tetrandrine (1–10 µM) (Sigma Aldrich), sunitinib (0.5–5 µM) (LC laboratories, Woburn, USA) and sorafenib (0.5–2 µM) (LC laboratories) and were also transferred after 24 hours to new plates with fresh medium containing the inhibitors. For mature fibrillar and total collagen detection after the addition of antifibrotic drugs, the highest concentration of the compounds was used. Stock solutions of the compounds were prepared in Milli-Q water or DMSO and diluted in the culture medium with a final concentration of the solvent of ≤1%.

### Viability

After incubation slices were transferred to 1 ml of a sonication solution, containing 70% ethanol and 2 mM EDTA, snap frozen in liquid nitrogen and stored at −80°C. To determine the viability, ATP levels were measured in the supernatant of samples homogenized for 45 sec in a Mini-BeadBeater-8 (Biospec, Bartlesville, OK, USA) and centrifuged for 2 min. at 16.000 g, using the ATP bioluminescence kit (Roche diagnostics, Mannheim, Germany) [27]. ATP values (pmol) were divided by the total protein content (µg) of the slice estimated by Lowry (Bio-Rad RC DC Protein Assay) [28]. Values for the control slices are expressed relative to the slices before incubation (t = 0). Values displayed for the antifibrotic compounds are relative values compared to the related controls incubated for 48 hours.

### Gene expression

To determine the antifibrotic effect of the compounds on the fPCLS, *Hsp47*, *αSma* and *Pcol1A1* gene expression was determined using Quantitative Real-Time PCR. First, the total RNA of three pooled snap-frozen slices was isolated using the RNeasy Mini Kit (Qiagen, Venlo, The Netherlands). The amount of isolated RNA was measured with the ND-1000 spectrophotometer (Fisher Scientific, Landsmeer, The Netherlands). Reverse transcriptase was performed with 1 µg RNA using the Reverse Transcription System (Promega, Leiden, The Netherlands). The RT-PCR reaction was performed in the Eppendorf mastercycler gradient at 25°C for 10 minutes, 45°C for 60 minutes and 95°C for 5 minutes. The gene expression of *Hsp47*, *αSma* and *Pcol1A1* was determined using the primers (50 µM) en probes (5 µM) listed in table 1 and the qPCR mastermix plus (Eurogentec, Maastricht, The Netherlands). The Real Time PCR reaction was performed in a 7900HT Real Time PCR (Applied Biosystems, Bleiswijk, The Netherlands) with 1 cycle of 10 minutes at 95°C and 45 cycles of 15 seconds at 95°C and 1 minute at 60°C.

**Table 1:** Primers and probes.

Primer/Probe	Sequence	Accession number
*Hsp47* Forward	5′-AGACGAGTTGTAGAGTCCAAGAGT-3′	
*Hsp47* Reverse	5′-ACCCATGTGTCTCAGGAACCT-3′	NM_017173
*Hsp47* Probe	5′-CTTCCCGCCATGCCAC-3′	
*αSma* Forward	5′-AGCTCTGGTGTGTGACAATGG-3′	
*αSma* Reverse	5′-GGAGCATCATCACCAGCAAAG-3′	BC158550
*αSma* Probe	5′-CCGCCTTACAGAGCC-3′	
*Pcol1A1* Forward	5′-CCCACCGGCCCTACTG-3′	
*Pcol1A1* Reverse	5′-GACCAGCTTCACCCTTAGCA-3′	NM_053304
*Pcol1A1* Probe	5′-CCTCCTGGCTTCCCTG-3′	
*Gapdh* Forward	5′-GAACATCATCCCTGCATCCA-3′	
*Gapdh* Reverse	5′-CCAGTGAGCTTCCCGTTCA-3′	XR_008524
*Gapdh* Probe	5′-CTTGCCCACAGCCTTGGCAGC-3′	

Ct values were corrected for the Ct values of the housekeeping gene *Gapdh* (ΔCt) and compared with those of slices before incubation (t = 0) for the untreated slices and for the slices treated with the antifibrotic compounds with the control slices incubated for 48 hours (ΔΔCt). Results are displayed as fold change (2^−ΔΔCt^).

### Collagen 1 protein expression analysis by western blotting

For the measurement of collagen 1 protein in fPCLS, 3 slices were pooled and snap frozen in liquid nitrogen and stored at −80°C until analysis. After thawing, the slice tissue was lysed for 1 hour on ice with RIPA buffer (1 protease inhibitor cocktail tablet (Boehringer Mannheim), in 250 µl of buffer pH 7.5, containing 50 mM Tris/HCl, 150 mM NaCl, 1% Igepal CA-630, 0.5% sodium deoxycholate, 0.1% SDS). The tissue was homogenized on ice by a Potter homogenizer and centrifuged for 1 hour at 4°C at 16.000 g. Protein concentrations were determined in the supernatant using a Bio-Rad DC protein assay according to the protocol. Lysates were diluted with 4 x SDS sample buffer (50 mM TrisHCl pH 6.8, 2% SDS, 10% Glycerol, 5% β-mercaptoethanol, 0.05% bromophenol blue) and boiled for 2 minutes. Tissue lysate (100 µg of protein) was size fractionated on a 7.5% Sodium Dodecyl Sulphate Poly Acrylamide gel by electrophoresis and transferred to an activated Polyvinylidene Difluoride membrane (Bio-Rad). After blocking for 1 hour in Tris buffered saline supplemented with 5% Blocking Grade Powder (Bio-Rad) and 0.1% Tween-20, immunodetection of collagen-1 (1∶1000, Rockland Immunochemicals) was performed. Binding of the antibody was determined using horseradish peroxidase conjugated secondary goat anti-rabbit and tertiary rabbit anti-goat antibody (Dako, Heverlee, Belgium). Visualization was performed with Western Lightning Plus-ECL (Perkin Elmer, Groningen, The Netherlands) and equal protein loading was confirmed by immunostaining with monoclonal anti B-actin (clone AC-74) (Sigma Aldrich Chemie B.V., Zwijndrecht, The Netherlands). The data of the untreated slices were expressed relative to the t = 0 value and the data of the slices treated with the antifibrotic compounds were expressed relative to that of slices incubated for 48 hours.

### Fibrillar collagen staining

Sirius red staining histochemistry was performed on 4 µm paraffin sections of formalin fixed fPCLS. Staining for fibrillar collagens was performed using the picrosirius red dye (Sigma, Gillingham, UK), which was quantitated using the Cell D computer program (Olympus, Hamburg, Germany). Each bar represents the average of the data of 3 different livers, and of each liver, 3 slices were stained per condition and of each slice the whole area of the section was analyzed for quantification. The data were expressed relative to that of control slices incubated for 48 hours.

### Total collagen assay by hydroxyproline measurement

After incubation, triplicate slices were pooled and snap-frozen in liquid nitrogen and stored at −80°C until analysis. After weighing the tissue, 100 µl of 6 M HCl was added and tissue was hydrolyzed at 95°C for 20 hours. Hydroxyproline was measured using the QuickZyme Total Collagen Assay (QuickZyme BioSciences, Leiden, The Netherlands) following the manufacturers protocol. Hydroxyproline content (µg) was corrected for the weight of the slices. The data of the untreated slices were expressed relative to the t = 0 value and the data of the slices treated with the antifibrotic compounds were expressed relative to that of control slices incubated for 48 hours.

### Statistics

A minimum of three different BDL-livers was used for each experiment, using slices in triplicate from each liver. As not all drugs could be tested in the same liver and for each liver control slices were included, the number of livers used for control slices is larger than the number of livers incubated with compounds. The results of the treatments were compared to the untreated controls using the Student's-t-test. The results are expressed as means ± SEM.

A *p*-value <0.05 was considered significant. Statistical differences were determined on relative ATP-, Western blot, Sirius red and hydroxyproline content values and ΔΔCt values of gene expression data.

## Results

### Non-treated fibrotic PCLS

**Figure 1:**
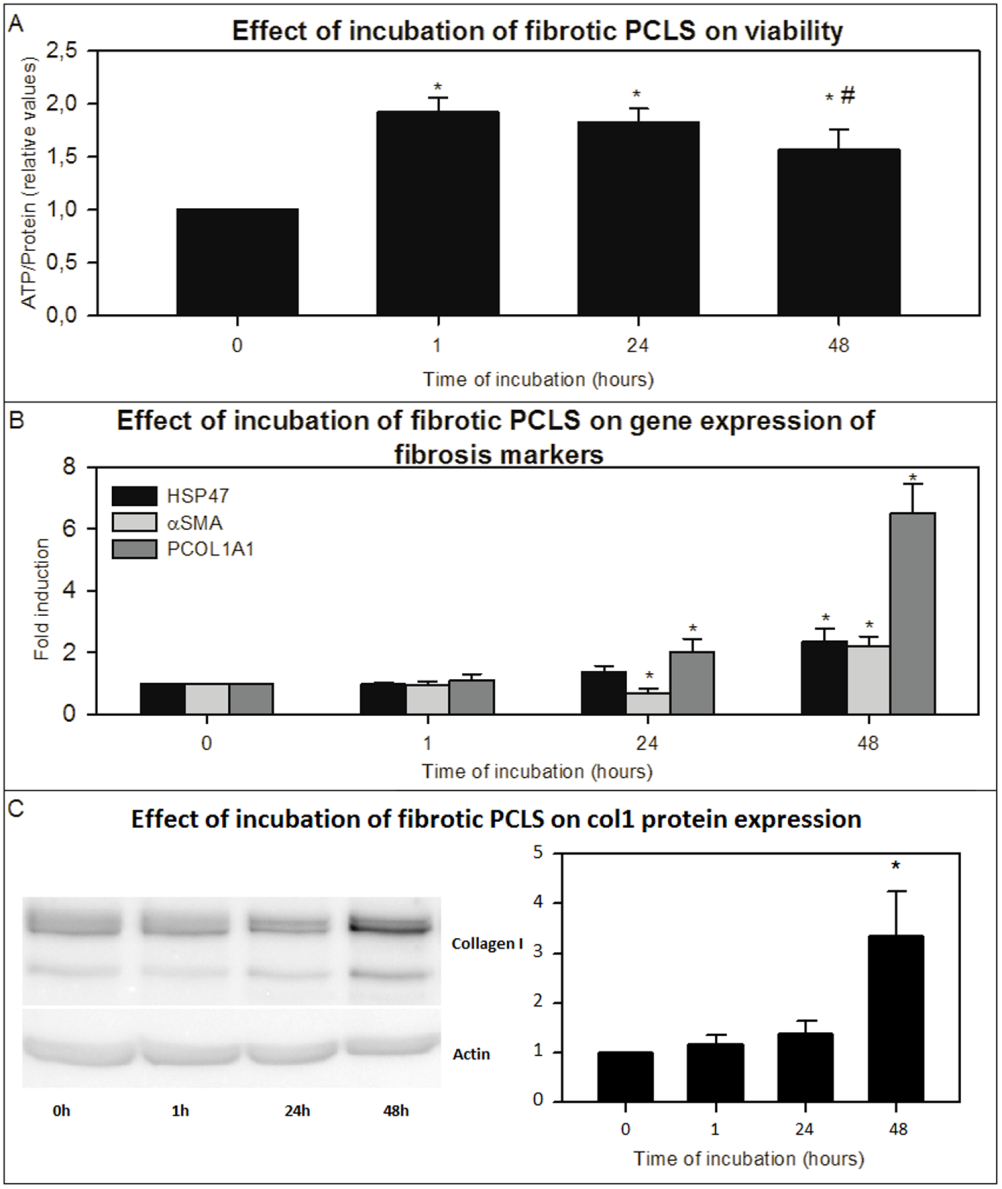
The effect of incubation of fPCLS on viability, gene expression of fibrosis markers and collagen 1 protein expression. The effect of incubation up to 48(A) (n = 12), on the gene expression of fibrosis markers *Hsp47*, *αSma* and *Pcol1A1* (B) (n = 13) and on the collagen 1 protein expression measured by western blot (pictures are from a representative experiment) (C) (n = 10) of fibrotic PCLS from livers of BDL rats. Error bars represent standard error of the mean (SEM). *p<0.05 vs 0 hours. #p<0.05 vs 1 hour.

**Figure 2:**
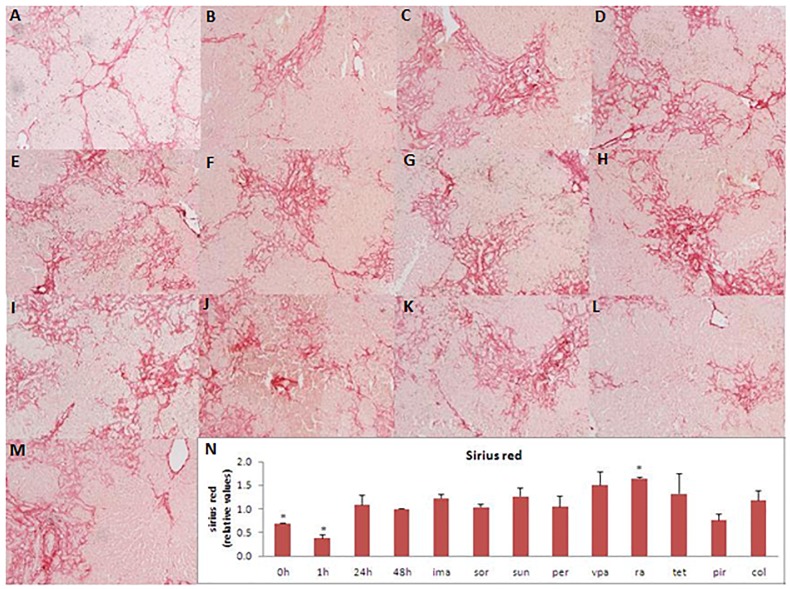
Sirius red staining of control fPCLS and fPCLS incubated with antifibrotic drugs. Sirius red staining on paraffin embedded, formalin fixed fibrotic PCLS directly after slicing (A), incubated for 1 hour (B), 24 hours (C) and 48 hours (D) and incubated for 48 hours with the compounds imatinib (ima) (E), sorafenib (sor) (F), sunitinib (sun) (G), perindopril (per) (H), valproic acid (vpa) (I), rosmarinic acid (ra) (J), tetrandrine (tet) (K), pirfenidone (pir) (L) and colchicine (col) (M).Quantification of sirius red staining (N). n = 3. Error bars represent SEM. *p<0.05 vs 48 hours.

**Figure 3:**
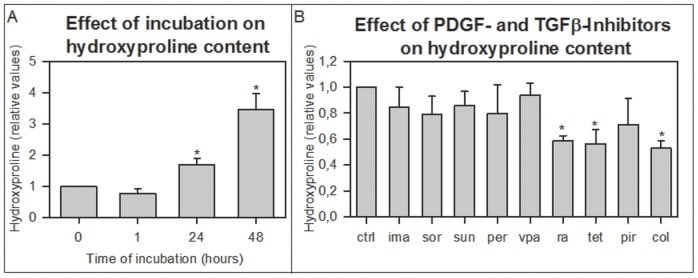
Hydroxyproline content of control fPCLS and fPCLS incubated with antifibrotic drugs. Hydroxyproline content in fPCLS incubated up to 48(A) and with addition of 10 µM imatinib (ima), 2 µM sorafenib (sor), 5 µM sunitinib (sun), 100 µM perindopril (per), 1 mM valproic acid (vpa) 270 µM rosmarinic acid (ra), 100 µM tetrandrine (tet), 2.5 mM perindopril (pir) and 200 nM colchicine (col) (B). n = 3. Error bars represent SEM. *p<0.05 vs 0h (A), *p<0.05 vs ctrl 48h (B).

Slices from BDL-livers showed increased ATP levels after 1 hour of incubation reflecting the recovery of the ATP levels after the period of cold preservation and slicing. The ATP levels remained constant until 24 hours and were slightly decreased after 48 hours (18% when compared to the 1 hour control) but were not significantly different from 24 hours samples (fig. 1A). In PCLS from BDL-livers directly after preparation, gene-expression of fibrosis markers *Hsp47*, *αSma* and *Pcol1A1* were significantly higher than those in PCLS from healthy rat livers (data not shown). Incubation of the fibrotic liver slices from the BDL rats was accompanied by a further increase of the gene expression of fibrosis markers after 48 hours compared to slices directly after slicing (fig. 1B). Already after 24 hours of incubation the *Pcol1A1* gene-expression was significantly elevated compared to fPCLS directly after preparation (fig. 1B). Furthermore, after 48 hours, also the protein expression of collagen 1 was increased compared to freshly prepared slices (fig. 1C). Sirius red staining and hydroxyproline content of the slices were increased after 24 and 48 hours of incubation (fig. 2A-D,N and fig. 3A).

### Effect of PDGF-inhibitors on fibrotic PCLS

**Figure 4:**
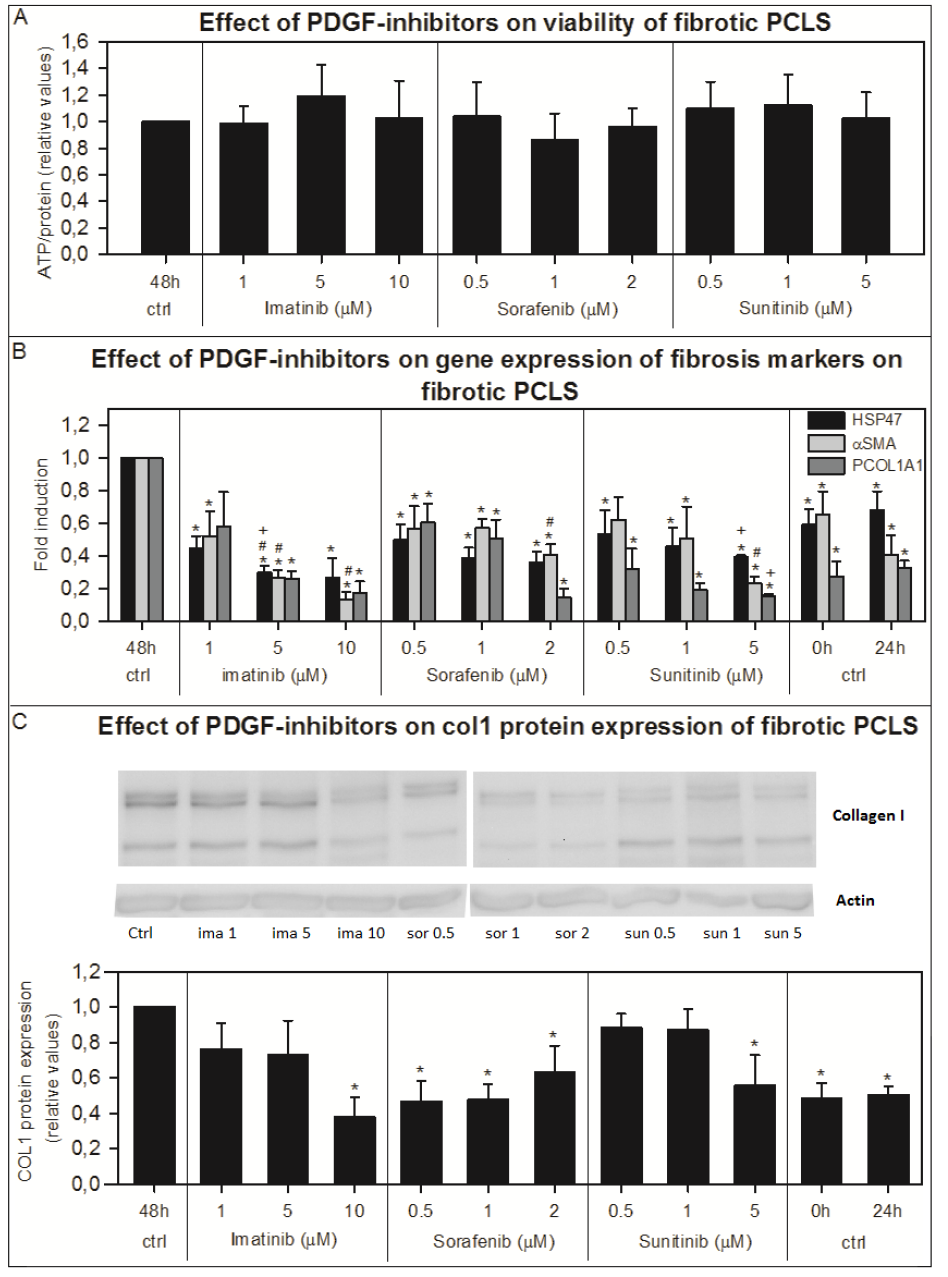
The effect of PDGF-inhibitors in fPCLS on viability, gene expression of fibrosis markers and collagen 1 protein expression. The effect of PDGF-inhibitors imatinib (n = 4), sorafenib (n = 4) and sunitinib (n = 4) on the viability (A), on the gene expression of fibrosis markers *Hsp47*, *αSma* and *Pcol1A1* (B) and on the collagen 1 protein expression (pictures are from a representative experiment) (C) of fibrotic PCLS from livers from BDL rats incubated for 48 hours. Error bars represent SEM. Significant decrease: *p<0.05 vs 48h, #p<0.05 vs 0 hours, +p<0.05 vs 24 hours.

The ATP content of the fPCLS indicated that the different concentrations of imatinib, sorafenib and sunitinib used in this study, were not toxic in fPCLS from BDL rats (fig. 4A). Incubation for 48 hours with increasing concentrations of these inhibitors showed a significant concentration dependent decrease in the gene expression of fibrosis markers *Hsp47*, *αSma* and *Pcol1A1* compared to 48 hours incubated control slices (fig. 4B). The gene expression of *Hsp47* in fPCLS, incubated for 48 hours with 5 µM of imatinib, was even significantly lower than in the control fPCLS directly after slicing and fPCLS incubated for 24 hours. Also, the *αSma* gene expression of fPCLS incubated with 5 and 10 µM imatinib, 2 µM sorafenib and 5 µM sunitinib was significantly lower than that of control fPCLS directly after slicing (fig. 4B). Moreover, in fibrotic PCLS incubated for 48 hours in the presence of 5 µM sunitinib, the gene expression of *Hsp47* and *Pcol1A1* was significantly lower than that of control fPCLS incubated for 24 hours (fig. 2B). Collagen 1 protein expression was also significantly decreased after 48 hours of incubation with the PDGF-inhibitors compared to the non-treated fibrotic slices (fig. 4C), while the Sirius red staining and the hydroxyproline content were not changed compared to control incubation (fig. 2 and fig. 3).

### Effect of TGFβ-inhibitors on fibrotic PCLS

**Figure 5:**
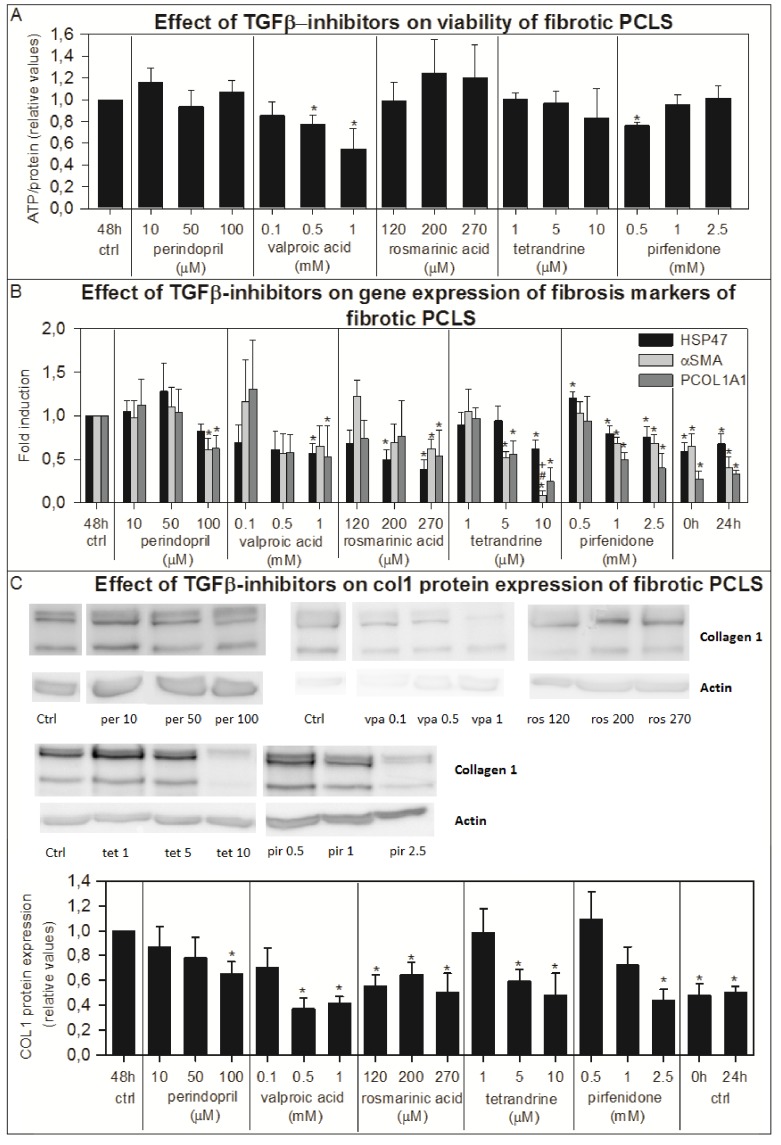
The effect of TGFβ-inhibitors in fPCLS on viability, gene expression of fibrosis markers and collagen 1 protein expression. The effect of TGFβ-inhibitors perindopril (n = 5), valproic acid (n = 4), rosmarinic acid (n = 4), tetrandrine (n = 4) and pirfenidone (n = 5) on the viability (A), on the gene expression of fibrosis markers *Hsp47*, *αSma* and *Pcol1A1* (B) and on the collagen 1 protein expression (pictures are from a representative experiment) (C) of fPCLS from livers from BDL rats incubated for 48 hours. Error bars represent SEM. Significant decrease: *p<0.05 vs 48h, #p<0.05 vs 0 hours, +p<0.05 vs 24 hours.

Perindopril, rosmarinic acid and tetrandrine had no effect on ATP values of fPCLS, however, pirfenidone (0.5 mM) decreased viability by 24% although only at the lowest concentration and valproic acid showed a concentration dependent decrease in ATP values up to maximally 45% at 1 mM compared to control fPCLS (fig. 5A). All TGFβ-inhibitors showed a concentration dependent reduction of the gene expression of the 3 fibrosis markers (fig. 5B), while 10 µM tetrandrine even decreased the *αSma* gene expression to a level lower than that of control slices directly after sling and control slices incubated for 24 hours (fig. 5B). On the contrary, 0.5 mM pirfenidone increased the gene expression of *Hsp47* by 20% in fPCLS. All TGFβ-inhibitors caused a decrease in collagen 1 protein expression (fig. 5C). However, the fibrillar collagen content as detected by the Sirius red staining was not decreased by the TGFβ inhibitors and rosmarinic acid even slightly increased the Sirius red staining compared to control fPCLS (fig. 2). The total collagen content as detected by the hydroxyproline content was only decreased after addition of rosmarinic acid and tetrandrine (fig. 3).

### Effect of Colchicine on fibrotic PCLS

**Figure 6:**
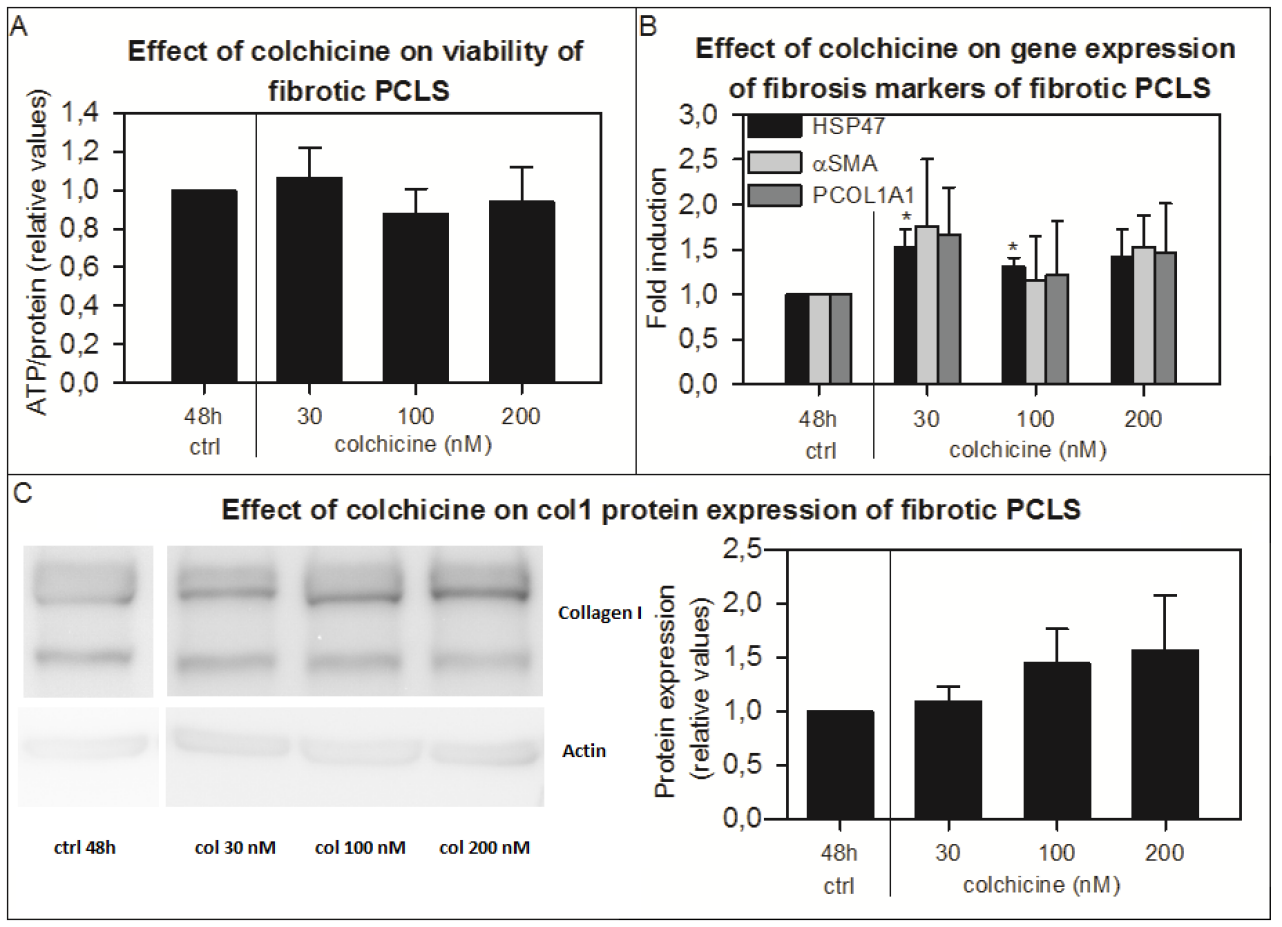
The effect of colchcine in fPCLS on viability, gene expression of fibrosis markers and collagen 1 protein expression. The effect of colchicine (n = 4) on the viability (A), on the gene expression of fibrosis markers *Hsp47*, *αSma* and *Pcol1A1* (B) and on the collagen 1 protein expression (pictures are from a representative experiment) (C) of fPCLS from livers from BDL rats incubated for 48 hours. Error bars represent SEM. *p<0.05 vs 48h.

Colchicine had no effect on the viability of fPCLS from BDL rats during 48 hours of incubation (fig. 6A). Concentrations of 30 nM and 100 nM significantly increased the gene expression of *Hsp47* (fig. 6B), while *αSma* and *Pcol1A1* gene expression, Sirius red staining and collagen 1 protein expression were not changed after incubation with colchicine (fig. 2 and 6). However, hydroxyproline content was decreased after addition of colchicine (fig. 3).

## Discussion

Recently, we showed that PCLS from healthy rat livers can be used to study the early onset of liver fibrosis [14]. In this current study we investigated whether this *ex vivo* model could also be utilized to study the end-stage of liver fibrosis, by using fibrotic rat PCLS. Incubation of fPCLS prepared from BDL rat livers resulted in an increase of fibrosis markers in the liver slices after 48 hours. Hence, although fPCLS directly after slicing of a BDL liver are already fibrotic, during incubation an even further increase of gene expression of *Hsp47*, *αSma*, *Pcol1A1,* protein expression of collagen 1, fibrillar collagen and total collagen deposition was observed, suggesting that further HSC activation and fibrogenesis occurs, as was shown before [15]. The initial decrease in *αSma* gene expression at 24 hours might be explained by an initial improvement due to the termination of the bile duct ligation or loss of fibroblasts and again an activation of HSC due to long-term incubation.

In this study we investigated the effect of a series of compounds predominantly inhibiting the TGFβ pathway (TGFβ-inhibitors) or the PDGF pathway (PDGF-inhibitors).

The PDGF-inhibitors imatinib, sorafenib and sunitinib clearly showed antifibrotic effects as they significantly decreased the gene expression of *Hsp47*, *αSma* and *Pcol1A1* in fPCLS after 48 hours of incubation. These compounds also showed antifibrotic effects in the model of early onset of fibrosis in PCLS [14]. Moreover these data confirm the data of Van de Bovenkamp *et al.* who also showed a decrease in *Pcol1A1* and *αSma* gene expression in fPCLS from BDL rats after 24 hours of incubation with imatinib compared to control slices without imatinib [15]. Collagen 1 protein expression was also decreased after 48 hours of incubation, however the PDGF-inhibitors did not decrease the fibrillar collagen and total collagen levels. Suggesting that the decrease in collagen 1 is not large enough to decrease the total collagen content. In the present study the gene expression of *αSma* after 48 hours of incubation of fPCLS with 5 µM imatinib was even lower than in the 24 hours control slices, suggesting that imatinib not only inhibited the increase of the fibrosis marker which occurred between 24 hours and 48 hours of culture, but even further reduced the *αSma* expression. This result is in line with *in vivo* studies where imatinib caused a reduction in the number of proliferating HSC and αSma positive cells 48 hours after BDL surgery in rats [29]. In contrast to the findings with fPCLS, *in vivo* this was accompanied by a reduced Sirius red staining for fibrillar collagen [29], which may be explained by the differences in experimental conditions, as in our studies imatinib was given for 48 hours, 3 weeks after BDL surgery, while *in vivo* imatinib was administered 1 day prior to BDL and treatment was continued for 3 days. This time dependency was also observed in BDL rats *in vivo* where a reduction in fibrillar collagen, assessed by an aniline blue staining, by imatinib was observed during the first 21 days after BDL, but not when administered during 22–35 days after BDL [30]. To our knowledge hydroxyproline has not been measured before after imatinib treatment in BDL rats, but it was decreased after imatinib treatment in a rat model of pig-serum induced fibrosis [16]. However in the latter study imatinib was administered directly at the start of the treatment with pig-serum [16] when fibrosis was not yet established, which might explain the different results of hydroxyproline in fPCLS.

Sorafenib, reduced the gene expression of fibrosis markers in the *ex vivo* fPCLS in accordance with results obtained in rats *in vivo* [31], [32]. However, Sirius red was not reduced in fPCLS whereas *in vivo* reduced Sirius red staining was observed after two weeks treatment with sorafenib, starting two weeks after BDL [31], which may indicate that the lack of effect on fibrillar collagen in our studies is due to the relatively short duration of the treatment. Wang *et al.* reported a reduction in both collagen 1 and hydroxyproline content in BDL rats treated with sorafenib during week 3 and 4 of BDL [17], while in fPCLS collagen 1 was reduced, but hydroxyproline content was not, which again may be explained by the short duration of treatment in the fPCLS studies.

To date, sunitinib was not tested *in vivo* in BDL rats, however it was investigated *in vivo* in a model of CCl_4_-induced fibrosis [18]. Although the pathophysiology of these two fibrosis models shows some differences, the effect of the PDGF-inhibitor imatinib was similar in both models [29], [33]. Sunitinib reduced the collagen deposition (Masson's trichrome staining) and the gene expression of *αSma* and collagens in CCl_4_ treated rats [18]. The antifibrotic *ex vivo* effects of sunitinib are therefore in line with these *in vivo* results with respect to the fibrosis markers and the collagen 1 deposition. The lack of effect of sunitinib on the fibrillar and total collagen deposition in the fPCLS could not be compared with *in vivo* data as they are not available.

To summarize, all tyrosine kinase inhibitors reduced the collagen 1 protein and gene expression of fibrosis markers after 48 hours of incubation in fPCLS compared to control fPCLS incubated for 48 hours, however the fibrosis markers were not reduced compared to control slices incubated for 1 hour. Thus, this implies that the antifibrotic compounds inhibited the increase of fibrosis markers during incubation. In addition, PDGF-inhibitors had an effect on collagen 1 only and not on fibrillar and total collagen levels, probably due to the short durations of treatment in the fPCLS studies.

To investigate if TGFβ-inhibitors were also effective in the fibrotic PCLS model, the effect of perindopril, valproic acid, rosmarinic acid, tetrandrine and pirfenidone was investigated. *Ex vivo* perindopril treatment decreased *αSma* and *Pcol1A1* gene expression and decreased the amount of collagen 1, in line with results obtained *in vivo* both in BDL induced as well as in CCl_4_ induced liver fibrosis [33], [34]. Similar to the findings with the PDGF-inhibitors also with perindopril the hydroxyproline content was not decreased in fPCLS, whereas it was decreased *in vivo* which might be explained by the longer treatment period of 21 days.

*In vivo* valproic acid was not investigated in BDL rats, however it was studied in CCl_4_ treated mice [20]. The antifibrotic effect of valproic acid was shown by a down-regulation of *αSma* and *Pcol1A1* in valproic acid treated CCl_4_ mice (valproic acid in drinking water starting 2 days before CCl_4_ injections) [20]. The *ex vivo* antifibrotic effect of valproic acid in rat fPCLS was comparable with these *in vivo* findings, despite the use of a different species, and was shown by a reduction in the amount of collagen 1, an inhibition of *Hsp47* and *Pcol1A1* expression and a tendency to reduce the *αSma* expression. Again a reduced Sirius red staining was observed *in vivo* after valproic acid treatment [20], and not in the fPCLS, possibly because of the different dosage regimen. The decreased viability of fibrotic PCLS after valproic acid administration was also found before in healthy rat and pig PCLS [14], [35], which may have affected the results. Moreover valproic acid is known to have hepatotoxic effects in mice *in vivo* [36]. However, as RNA could be isolated and the expression of the housekeeping gene was constant in time we included the 1 mM valproic acid.

Also rosmarinic acid was not investigated before in a BDL rat fibrosis model, but was investigated in a CCl_4_ rat model (6 weeks) for stationary liver fibrosis [21]. As in our fPCLS, hydroxyproline content was reduced in CCl_4_ rats treated with rosmarinic acid [21]. The reason why fibrillar collagen was slightly increased in fibrotic PCLS after treatment with rosmarinic acid needs to be further investigated, as no papers about the effect of rosmarinic acid on sirius red in liver fibrosis have been published yet.

A reduced amount of collagen 1, hydroxyproline content and *αSma*, *Hsp47* and *Pcol1A1* was found in fPCLS after tetrandrine addition, which is comparable with *in vivo* studies in BDL rats [37], [38]. Treatment of BDL rats with tetrandrine directly after surgery, reduced the number of αSma positive cells [37], [38], Sirius red staining [37] hydroxyproline content [38] and the gene expression of *αSma* and *Col1A2* [37]. However, in the fPCLS model, tetrandrine was added when the slices were already fibrotic, which might explain the fact that Sirius red was not reduced after tetrandrine treatment, as it can be assumed that the formation of fibrillar collagen occurs after the synthesis of collagen.

Like the other TGFβ-inhibitors, studies with pirfenidone showed comparable results *in vivo* and *ex vivo*. Pirfenidone treatment was investigated *in vivo* in 4 weeks BDL rats, administered directly after BDL surgery [23] and 2 weeks after BDL surgery [39]. A reduction in Sirius red staining and hydroxyproline content [23] and gene expression of *Col1A* [39] after treatment with pirfenidone was seen.

In conclusion, all five TGFβ-inhibitors showed antifibrotic effects in fibrotic rat PCLS which was comparable with the *in vivo* effects of these compounds on liver fibrosis. Both *Pcol1A1* gene expression and collagen 1 protein expression was decreased after addition of the TGFβ-inhibitors. However, fibrillar collagen levels measured by Sirius red were not decreased and hydroxyproline, a measurement of total collagen was only decreased in slices treated with rosmarinic acid and tetrandrine, indicating that these compounds may also have effects on other collagens. In addition, the effect of TGFβ-inhibitors on stationary fibrosis is different from the effect of these inhibitors on the early onset of liver fibrosis in PCLS [14]. TGFβ-inhibitors only slightly reduced the fibrosis markers in the early onset of fibrosis in PCLS [14], which suggests that the TGFβ pathway plays a minor role in the early onset of liver fibrosis in PCLS and a larger role in end-stage liver fibrosis in fPCLS.

Colchicine slows down the microtubule mediated transport of procollagen [40], which may explain its antifibrotic effect as observed in BDL rats where colchicine reduced the connective tissue volume (Masson's trichrome staining) [41], reduced the increased collagen deposition (trichrome staining) [42], [43] and the hydroxyproline content [43]. Also in the *ex vivo* model of fPCLS, colchicine induced a decrease in total collagen content although fibrillar collagens were not yet changed.

In this study the effect of the antifibrotic drugs on the collagen protein content was assessed on three different levels, the amount of collagen 1 (by western blotting), the total collagen content (by hydroxyproline determination) and the amount of fibrillar collagens (by Sirius red staining). Both PDGF-inhibitors and TGFβ-inhibitors reduced the collagen 1 deposition, while total collagen was only decreased by the TGFβ-inhibitors rosmarinic acid and tetrandrine and fibrillar collagens (mature collagen) were not changed by any of the drugs. Apparently collagen 1 synthesis is primarily affected by these drugs, which is not yet detected by Sirius red or hydroxyproline content as mature fibrillar collagens do not only consist of collagen type I, but also type II, III, V and XI [44] and with a hydroxyproline measurement all collagens are measured [45]. Moreover reduction in mature fibrillar and total collagen is not only dependent on the synthesis of collagen but also on the rate of collagen breakdown, and therefore it may take more than 48 hours for an inhibition of synthesis to become manifest. From the results of this study it can be concluded that collagen 1 detection by western blot is the most sensitive method for the detection of antifibrotic effects on collagen disposition in fibrotic PCLS.

In conclusion, PCLS from fibrotic livers from rats 3 weeks after BDL can be successfully used as a functional *ex vivo* model to study molecular pathways in established liver fibrosis, as these fPCLS remain viable and fibrosis markers continue to increase during incubation. In addition, the *in vivo* observed antifibrotic effects of drugs acting on the PDGF- and TGFβ signaling pathway can successfully be reproduced *ex vivo* on the gene expression of fibrosis related genes and collagen I protein expression, indicating that this *ex vivo* model is a useful model for preclinical studies to test the effect of potential new antifibrotic drugs on these fibrosis markers. This will increase the efficiency of these studies as dose response studies of several drugs can be performed simultaneously with a limited number of rats, which also greatly reduces the number of laboratory animals utilized in fibrosis research. Further studies are ongoing to investigate the application of human PCLS from patients with liver fibrosis, which will be useful to identify species differences and to further reduce the use of experimental animals.
